# Deep learning-based recognition and segmentation of intracranial aneurysms under small sample size

**DOI:** 10.3389/fphys.2022.1084202

**Published:** 2022-12-19

**Authors:** Guangyu Zhu, Xueqi Luo, Tingting Yang, Li Cai, Joon Hock Yeo, Ge Yan, Jian Yang

**Affiliations:** ^1^ School of Energy and Power Engineering, Xi’an Jiaotong University, Xi’an, China; ^2^ Xi’an Key Laboratory of Scientific Computation and Applied Statistics, Xi’an, China; ^3^ School of Mathematics and Statistics, Northwestern Polytechnical University, Xi’an, China; ^4^ School of Mechanical and Aerospace Engineering, Nanyang Technological University, Singapore, Singapore; ^5^ Department of Radiology, The First Affiliated Hospital of Xi’an Jiaotong University, Xi’an, China

**Keywords:** SAH, intracranial aneurysm, automatic segmentation, convolutional neural network, deep learning

## Abstract

The manual identification and segmentation of intracranial aneurysms (IAs) involved in the 3D reconstruction procedure are labor-intensive and prone to human errors. To meet the demands for routine clinical management and large cohort studies of IAs, fast and accurate patient-specific IA reconstruction becomes a research Frontier. In this study, a deep-learning-based framework for IA identification and segmentation was developed, and the impacts of image pre-processing and convolutional neural network (CNN) architectures on the framework’s performance were investigated. Three-dimensional (3D) segmentation-dedicated architectures, including 3D UNet, VNet, and 3D Res-UNet were evaluated. The dataset used in this study included 101 sets of anonymized cranial computed tomography angiography (CTA) images with 140 IA cases. After the labeling and image pre-processing, a training set and test set containing 112 and 28 IA lesions were used to train and evaluate the convolutional neural network mentioned above. The performances of three convolutional neural networks were compared in terms of training performance, segmentation performance, and segmentation efficiency using multiple quantitative metrics. All the convolutional neural networks showed a non-zero voxel-wise recall (V-Recall) at the case level. Among them, 3D UNet exhibited a better overall segmentation performance under the relatively small sample size. The automatic segmentation results based on 3D UNet reached an average V-Recall of 0.797 ± 0.140 (3.5% and 17.3% higher than that of VNet and 3D Res-UNet), as well as an average dice similarity coefficient (DSC) of 0.818 ± 0.100, which was 4.1%, and 11.7% higher than VNet and 3D Res-UNet. Moreover, the average Hausdorff distance (HD) of the 3D UNet was 3.323 ± 3.212 voxels, which was 8.3% and 17.3% lower than that of VNet and 3D Res-UNet. The three-dimensional deviation analysis results also showed that the segmentations of 3D UNet had the smallest deviation with a max distance of +1.4760/−2.3854 mm, an average distance of 0.3480 mm, a standard deviation (STD) of 0.5978 mm, a root mean square (RMS) of 0.7269 mm. In addition, the average segmentation time (AST) of the 3D UNet was 0.053s, equal to that of 3D Res-UNet and 8.62% shorter than VNet. The results from this study suggested that the proposed deep learning framework integrated with 3D UNet can provide fast and accurate IA identification and segmentation.

## 1 Introduction

The intracranial aneurysm is the local abnormal bulge of the intracranial arterial wall, which occurs in 5%–8% of the general population (Schievink, 1997; Vlak et al., 2011; Cebral and Raschi, 2013). The IAs remain asymptomatic until rupture. The global incidence of subarachnoid hemorrhage (SAH) caused by an IA rupture varies from two to more than 20 per 100,000 persons-years, and the modality could be greater than 50% (Nam et al., 2015; Lawton and Vates, 2017; Etminan et al., 2019; Schatlo et al., 2021).

One of the major challenges in IAs management is rupture prediction (Wiebers et al., 1987; England, 1998; Rayz and Cohen-Gadol, 2020). In current clinical practices, rupture risk estimation of IAs mainly relies on morphological metrics, including size, location, aspect ratio (AR), and size ratio (Hademenos et al., 1998; Wardlaw and White, 2000; Lall et al., 2009; Ma et al., 2010; Duan et al., 2018). Thus, accurate measurement is the basis of successful prediction (Raghavan et al., 2005; Dhar et al., 2008; Zanaty et al., 2014; Leemans et al., 2019), especially for some sensitive parameters such as daughter sac and AR (Murayama et al., 2016; Wang et al., 2018). Traditionally only 2D information from neuroimaging was utilized in the interpreting and measuring IAs (Rayz and Cohen-Gadol, 2020), which neglected the complex 3D structure of IAs and may lead to measurement bias and inconsistency (Rajabzadeh-Oghaz et al., 2017, 2018). Studies have shown that morphological metrics derived based on 3D information are more accurate and consistent than 2D manual measurement (Ma et al., 2004; Ryu et al., 2011; Rajabzadeh-Oghaz et al., 2018). However, recent studies have revealed that the morphology metrics alone may not be sufficient for predicting the rupture risks of IA, especially in small unruptured IAs (Abboud et al., 2017; Korja et al., 2017; Longo et al., 2017; Hu et al., 2021; Ren et al., 2022).

In addition to morphological evaluation, hemodynamics’ role in IA rupture has drawn growing attention. Imaging-based patient-specific computational fluid dynamics (CFD) simulations have been regarded as a powerful tool for investigating the hemodynamics in the IAs (Xiang et al., 2011; Takao et al., 2012; Liang et al., 2016; Xu et al., 2018; Zhu et al., 2019a; Medero et al., 2020; Hu et al., 2021; Le, 2021; Li et al., 2022). Several quantitative hemodynamics metrics, such as average wall shear stress (WSS), maximum intra-aneurysmal WSS, low WSS area, average oscillatory shear index, and relative resident time, were identified to play a vital role in the pathologies of IA rupture. However, most metrics are derived from studies that only involve a single or relatively small volume of patients, which are statistically unconvincing. Moreover, the clinical guideline and practical scoring system that include the hemodynamics metrics for IA management are yet to be established. To overcome the problems mentioned above, single and multicenter studies that contain patient-specific hemodynamics analysis in larger cohorts would be required (Xiang et al., 2013; Ionita et al., 2014; Rayz and Cohen-Gadol, 2020).

Precise individualized 3D modeling of IA is the first and the most crucial step in the workflow of accurate patient-specific morphological and hemodynamics analyses. Conventionally, the IA recognition and segmentation in the modeling procedure mainly rely on manual operations. The manual detection and segmentation of IAs require researchers to have rich medical image interpretation experience (Firouzian et al., 2011; Sen et al., 2014; Yang et al., 2014; Kavur et al., 2020; Haider and Michahelles, 2021; Jalali et al., 2021). Due to the complexity of cerebrovascular anatomy, the procedure is error-prone, which could bring inconsistency in the modeling and induce errors in subsequent analyses (Sen et al., 2014; Schwenke et al., 2019; Bo et al., 2021; Mensah et al., 2022). In addition, the highly labor-intensive nature of the manual operations also prevents the application of patient-specific analyses in large cohorts. Thus, automating the modeling process has been a research Frontier.

With the development of machine learning in recent years, convolutional neural network (CNN) architecture has shown great potential in automatic medical image segmentation. Ronneberger et al. proposed the UNet, a U-shaped convolution neural network model (Ronneberger et al., 2015). This model can achieve accurate segmentation under a small dataset and is continuously applied, developed, and optimized. Based on UNet, deep residual UNet (Res-UNet) simplifies the training process of deep neural networks with a residual mechanism, achieving higher accuracy in aerial image-based road extraction (Zhang et al., 2018). VNet also adopts the residual mechanism based on UNet, showing excellent results in the field of prostate segmentation (Milletari et al., 2016). Based on these studies, many neural network models have emerged in the past 2 years to detect and segment IAs(Park et al., 2019; Shi et al., 2020a; Ma and Nie, 2021; Su et al., 2021). Park et al. (Park et al., 2019) developed a CNN model called HeadXNet, which can process the CTA images of patients and generate voxel-by-voxel prediction results and has passed the validation of the clinical application. Su et al. (Su et al., 2021) introduced the attention gate (AG) mechanism into the 3D UNet model to improve the performance of the UNet model under a small sample size. Ma et al. (Ma and Nie, 2021) adopted the 3D UNet model and configured it with a larger patch size to obtain more context information. The early experiences of CNN-assisted automatic IA segmentation have proved its practical value as a tool to assist clinical diagnosis and to improve the efficiency of the modeling procedure of IA (Zhao et al., 2018).

Furthermore, several groups have conducted studies to evaluate the segmentation performances between different CNN architectures. Karimov et al. (Karimov et al., 2019) compared the accuracy and performance of three CNNs (UNet, ENet, and BoxENet) for the segmentation of mast cells in scans of histological slices and found that UNet showed higher accuracy in terms of DSC, intersection over union (IoU) and F1-score. Kartali et al. (Kartali et al., 2018) compared three deep-learning approaches based on CNN and two conventional approaches for real-time emotion recognition of four basic emotions (happiness, sadness, anger, and fear) from facial images. Zhang et al. proposed the Dense-Dilated Neural Network (DDNet) based on 3D UNet for the segmentation of cerebral arteries in TOF-MRA images, which got better performance than UNet, Vnet, and Uception (Zhang and Chen, 2019). Zhu et al. (Zhu et al., 2019b) compared the segmentation performance of V-NAS, 3D UNet, and VNet on the dataset of both normal organs (NIH Pancreas) and abnormal organs (MSD Lung tumors and MSD Pancreas tumors). These studies suggested that the CNN architectures and the segmentation object could impact the segmentation performances. However, the performances of existing CNNs in IA segmentation are yet to be investigated.

In this study, we proposed a deep-learning-based segmentation framework for IA, and the impacts of pre-processing and convolutional neural network (CNN) architectures on IA segmentation performance were quantitively evaluated.

## 2 Methods

### 2.1 Data preparation

#### 2.1.1 Data collection

The Institutional Ethics Review Committee of the First Affiliated Hospital of Xi’an Jiaotong University approved this retrospective study. A dataset containing the CTA images of 101 patients with 140 IAs was retrospectively collected and fully anonymized from the First Affiliated Hospital of Xi’an Jiaotong University. The CTA images were captured by the 256-slice spiral CT scanners (BrillianceiCT, Philips Healthcare, Cleveland, OH, United States). The specific scanning parameters were as follows: tube voltage, 120 kV; tube current, 1,000 mA; layer thickness, 0.9 mm.

We included all CTA acquisitions with at least an aneurysm, irrespective of etiology, symptomatology, and configuration (saccular, fusiform, and dissecting). The aneurysms were located in the anterior cerebral arteries (ACA), the anterior communicating arteries (ACoA), internal carotid arteries (ICA), the middle cerebral arteries (MCA), the posterior cerebral arteries (PCA), and the vertebral basilar arteries (VA).

#### 2.1.2 Image annotation

The CTA images were annotated under the guidance of experienced clinicians. All aneurysms were manually segmented using the manual segmentation tool of ITK-SNAP. The location and diameter of the 140 IAs were determined and statistically classified.

#### 2.1.3 Dataset construction

**FIGURE 1:**
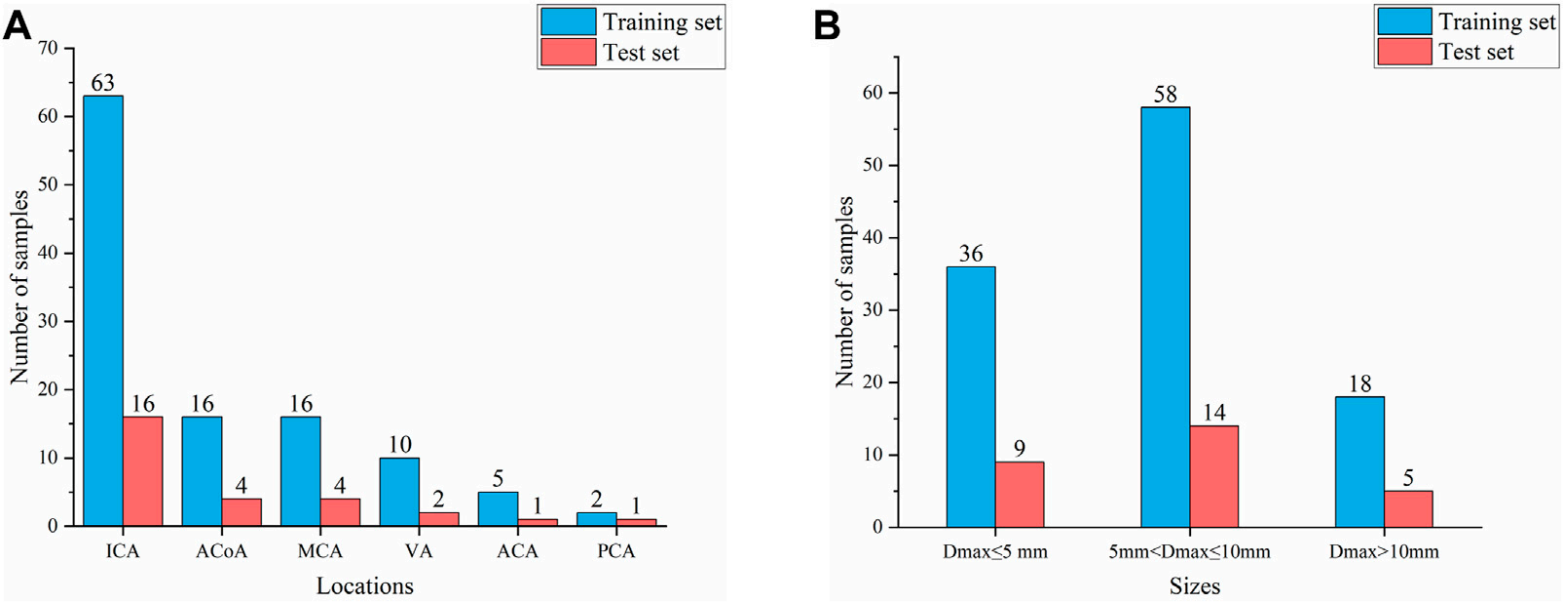
Composition of IAs of different locations and sizes **(A)** Aneurysm location distribution **(B)** Aneurysm size distribution.

According to the classification by location and size of IAs, 80% of cases were randomly divided as the training set, and the remaining 20% were used as the test set to ensure that the data distribution of the training set and the test set is as consistent as possible. Thus, the training set and test sets contained 112 and 28 aneurysms, respectively, as shown in Figure 1. Finally, 112 negative cases (no IAs occur) were added to the training set to balance the proportion of positive and negative samples. No validation set was set due to the small sample size in this study.

#### 2.1.4 Image pre-processing

**FIGURE 2:**
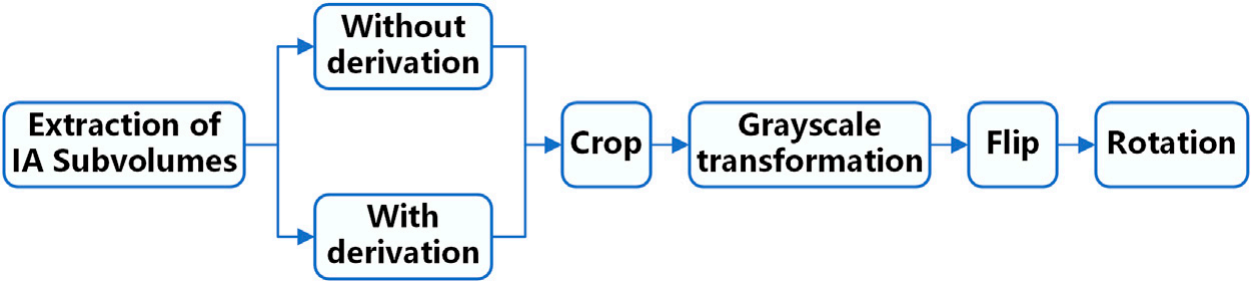
Data pre-processing process.

Before input to the network, the data needs to be pre-processed because the grey value of IA is relatively similar to the surrounding tissues and the lesion area occupies a relatively small proportion in the original image. Therefore, we set up a comparative experiment in this study. First, we performed first-order derivation on the image in advance to emphasize the boundary features of the aneurysm and performed the same follow-up pre-processing on the derivated and underived images. Then we input the derivated and underived images into the network model for training, respectively, and compared the segmentation effects of the models in the two situations to explore the sensitivity of the edge information to the deep learning network model. The data pre-processing process is shown in Figure 2.

In the above-mentioned follow-up pre-processing process, we first cropped the image into 48 × 48 × 48 voxel sub-volumes, thereby increasing the proportion of lesions in the image. We also performed a grayscale transformation to enhance the contrast between IA and the background region. After these two steps, the image features of the lesion are directly enhanced. Considering the small sample size of the dataset in this study, random flip and random rotation are also used for data augmentation in the pre-processing stage.

To explore the influence of different patch sizes on the automatic segmentation results of the model, this study set three different patch sizes, namely 32 × 32 × 32, 48 × 48 × 48, and 64 × 64 × 64 voxels.

### 2.2 Convolutional neural network

Convolutional neural network (CNN) is one of the most representative algorithms of deep learning, proposed firstly by Lecun et al. for image processing (LeCun et al., 1998). CNN usually consists of an input layer, multiple convolution layers, pooling layers, and fully connected layers. It uses a convolution kernel to extract features from the image and uses image filling strategy to retain the original image information as much as possible. Therefore, it can extract high-order features from input information, which is widely used in image recognition, target segmentation, natural language processing, and other fields. We built and compared three popular CNN models for medical image processing in this paper, including 3D UNet, VNet, and 3D Res-UNet.

#### 2.2.1 3D UNet

**FIGURE 3:**
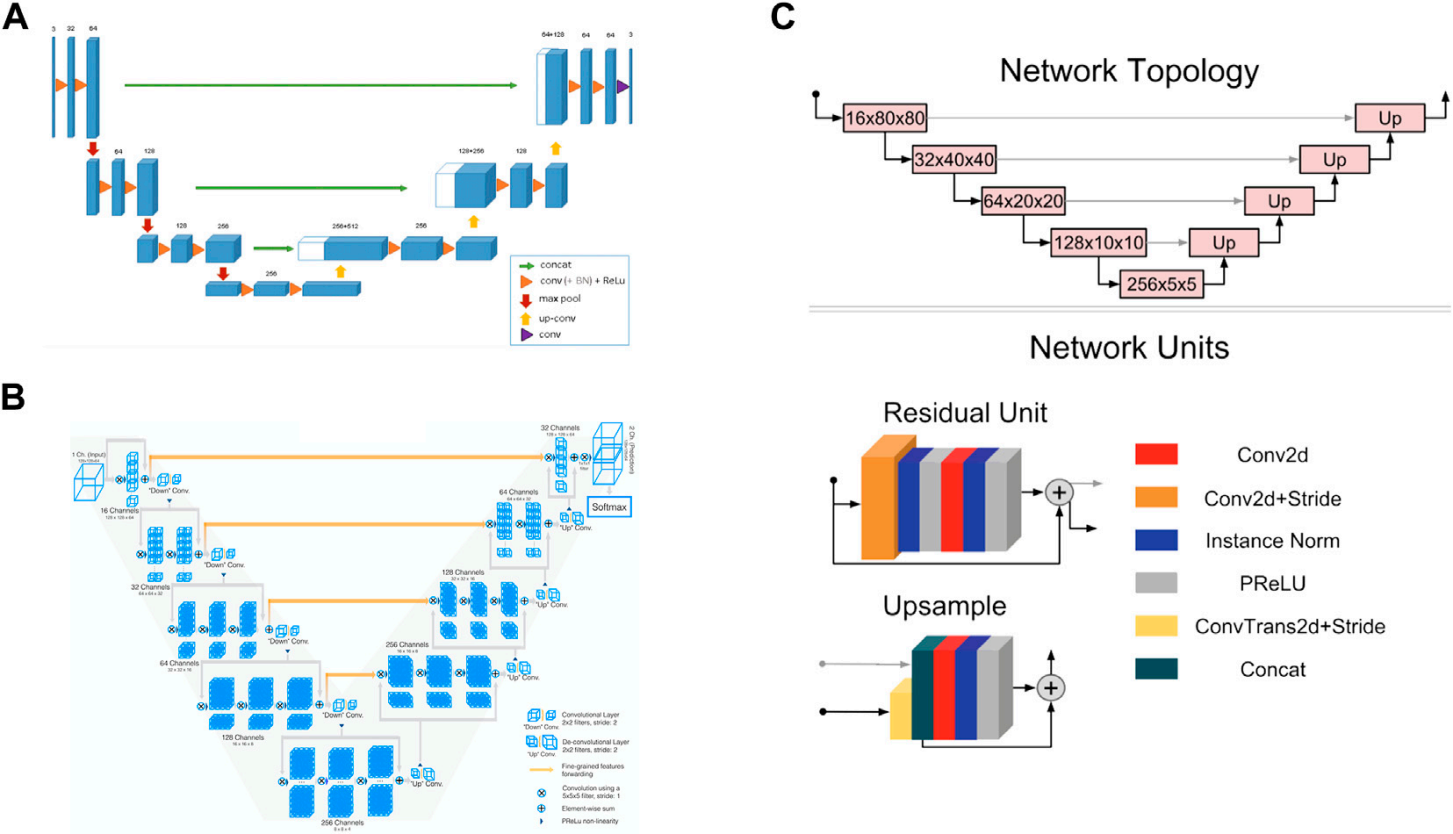
Convolutional neural network structures of **(A)** 3D UNet (Cicek et al., 2016) **(B)** VNet (Milletari et al., 2016), and **(C)** 3D Res-UNet (Kerfoot et al., 2019).

3D UNet is a CNN composed of a contracting path and an expansive path (Cicek et al., 2016). The contracting path is used to obtain context information, while the expansive path is used to locate accurately. They are almost symmetrical, forming a U-shaped network structure (Figure 3A).

In the encoder part of the left half, the downsampling module is repeatedly applied, which is composed of two 3 × 3 × 3 convolutions, each followed by a rectified linear unit (ReLU) and a 2 × 2 × 2 maximum pooling operation. After each pooling operation, the image size is reduced by one time, and the number of channels of the feature map is doubled.

In the decoder part of the right half, the upsampling module is repeatedly applied, which is composed of a 2 × 2 × 2 up-convolution and two 3 × 3 × 3 convolutions, each followed by a ReLU. In this process, the image size can be doubled by the 2 × 2 × 2 up-convolution operation, and the number of channels of the feature map can be doubled. Subsequently, the feature map obtained in the upsampling process and the corresponding cropped feature map in the contracting path are concatenated through skip connections so that more information can be integrated for more precise pixel positioning. In the last layer, a 1 × 1 × 1 convolutional layer is also added to reduce the number of channels of the output image to the number of labels.

3D UNet can accept images of any size because it does not contain full connection layers. In addition, 3D UNet uses batch normalization (BN) before each RELU to speed up convergence and avoid network structure bottlenecks.

#### 2.2.2 VNet

VNet is a CNN proposed for 3D medical image segmentation (Milletari et al., 2016). Similar to UNet, its contracting path and expansive path are almost symmetrical, forming a V-shaped network structure (Figure 3B).

The contracting path on the left is divided into different stages, including one to three convolutional layers, and each stage uses a convolution kernel with a size of 5 × 5 × 5 voxels for convolution operation. VNet introduces the residual function, which connects the feature map after convolution operation and PReLU non-linearity with the original input of this stage for element-wise residual connection. Then the convolution with 2 × 2 × 2 voxels wide kernels applied with stride two is performed for downsampling. Therefore, after each downsampling operation, the size of the feature map is reduced by half, and the number of channels is doubled.

The expansive path on the right continuously extracts features during the up-sampling process and increases the spatial support for lower-resolution feature maps to collect important feature information. Finally, softmax is used to generate probability distributions to achieve voxel-by-voxel classification.

Like UNet, VNet also transfers and superimposes the feature map of the contracting path on the left to the expansive path on the right through skip connections, supplementing the detailed information of the loss to improve the segmentation accuracy.

#### 2.2.3 3D Res-UNet

3D Res-UNet is a CNN model implemented by adding residual units based on UNet, as seen in Figure 3C (Kerfoot et al., 2019).

3D Res-UNet uses convolution and deconvolution with stride two to perform downsampling and upsampling operations instead of pooling layers so that the network can learn the best upsampling or downsampling operation and further reduce the number of network layers. In addition, parametric rectifying linear units (PReLU) are used in the residual unit to enable better activation of the network learning and improve the segmentation effect. Instance normalization is used to prevent contrast shift.

### 2.3 Training procedure

In this study, the three CNN models are constructed based on PyTorch (Paszke et al., 2019) and Monai (MONAI Consortium, 2020) deep learning frameworks. All training and testing tasks were carried out on the same deep learning platform and accelerated by GeForce GTX 1080 Ti GPU with 10 GB of memory. Each model was trained for 500 epochs using the Adam optimizer with an initial learning rate of 0.0001. As the criterion for convergence of model training, the Dice coefficient loss function is defined as Eq. 1, where *y*_*gt*_ and *y*_*pred*_ are the ground truth and binary predictions from the neural networks, respectively. Additionally, *ε* is an infinite decimal, set to 1e-5 here.

$$D_{loss}=1-\frac{\;2\left|y_{gt}\cap y_{pred}\right|+\varepsilon }{\left|y_{gt}\right|+\left|y_{pred}\right|+\varepsilon }$$ (1)

### 2.4 Performance evaluation

In this paper, we used V-Recall, DSC, HD, and AST to comprehensively evaluate the segmentation performance of three CNN models. Among them, V-Recall was used to characterize the voxel-wise accuracy of lesion recognition. DSC and HD were used to characterize the quality of lesion segmentation, and AST was used to characterize segmentation efficiency.

V-Recall: V-Recall is the voxel-wise ratio of the number of true positive IA voxels to the number of all true IA voxels in an IA lesion, which is defined as follows:

$$V-Recall=\frac{\left|y_{gt}\cap y_{pred}\right|}{\left|y_{gt}\right|}$$ (2)

where *y*_*gt*_ and *y*_*pred*_ represent the ground truth and the binary predictions from the neural networks and | *y*_*gt*_∩*y*_*pred*_ | is the intersection of the ground truth and the prediction, representing the predicted correctly lesion voxels. We used V-Recall to evaluate the ability of the model to accurately identify true IA voxels in an IA lesion since a segmentation task can be seen as a voxel-wise prediction. A non-zero V-Recall indicates that the lesion can be identified at the case level. The closer V-Recall is to 1, the more complete the segmentation of the lesion.

DSC: DSC represents the overlap ratio between the ground truth and segmentation results. Its value range is between 0 and 1, the closer to 1, the better the segmentation effect. DSC is defined as follows:

$$DSC=\frac{2\left|y_{gt}\cap y_{pred}\right|}{\left|y_{gt}\right|+\left|y_{pred}\right|}$$ (3)

HD: The HD measures the distance between the two point sets, representing the similarity of the two sets. The HD is sensitive to the boundary of the segmentation results. When the segmentation results predicted by the neural networks are closer to the ground truth, the HD is smaller. The HD between the two sets is defined as follows:

$$HD=\max\left\{d_{y_{gt}y_{pred}},d_{y_{pred}y_{gt}}\right\}=\max\{\max_{x\in y_{gt}}\min_{y\in y_{pred}}d\left(x,y\right),\max_{y\in y_{pred}}\underset{x\in y_{gt}}{\min\;}d\left(x,y\right)\}$$ (4)

where *d(x, y)* is the distance between point *x* of the ground truth (*y*_*gt*_) and point *y* of the predictions (*y*_*pred*_).

AST: AST is the average segmentation time consuming for a trained model to segment each sample, representing a trained model’s segmentation efficiency.

Because the above metrics (DSC, HD, V-Recall, *etc.*) cannot reflect the bias of geometric details, which might affect the accuracy of subsequent mechanical analysis, a 3D deviation analysis between CNN-based segmentation and ground truth was carried out in Geomagic Studio 2014 software (Raindrop Geomagic, Development Triangle, NC, United States). The 3D models were reconstructed from segmentation and ground truth based on the Python platform and VTK library (Schroeder et al., 2006). Furthermore, the geometric quality was evaluated using four metrics widely used in 3D model deviation analysis, including maximum distance, average distance, standard deviation (STD), and root mean square (RMS) value.

## 3 Results

### 3.1 impact of pre-processing method on model performance

#### 3.1.1 Impact of image derivation

**FIGURE 4:**
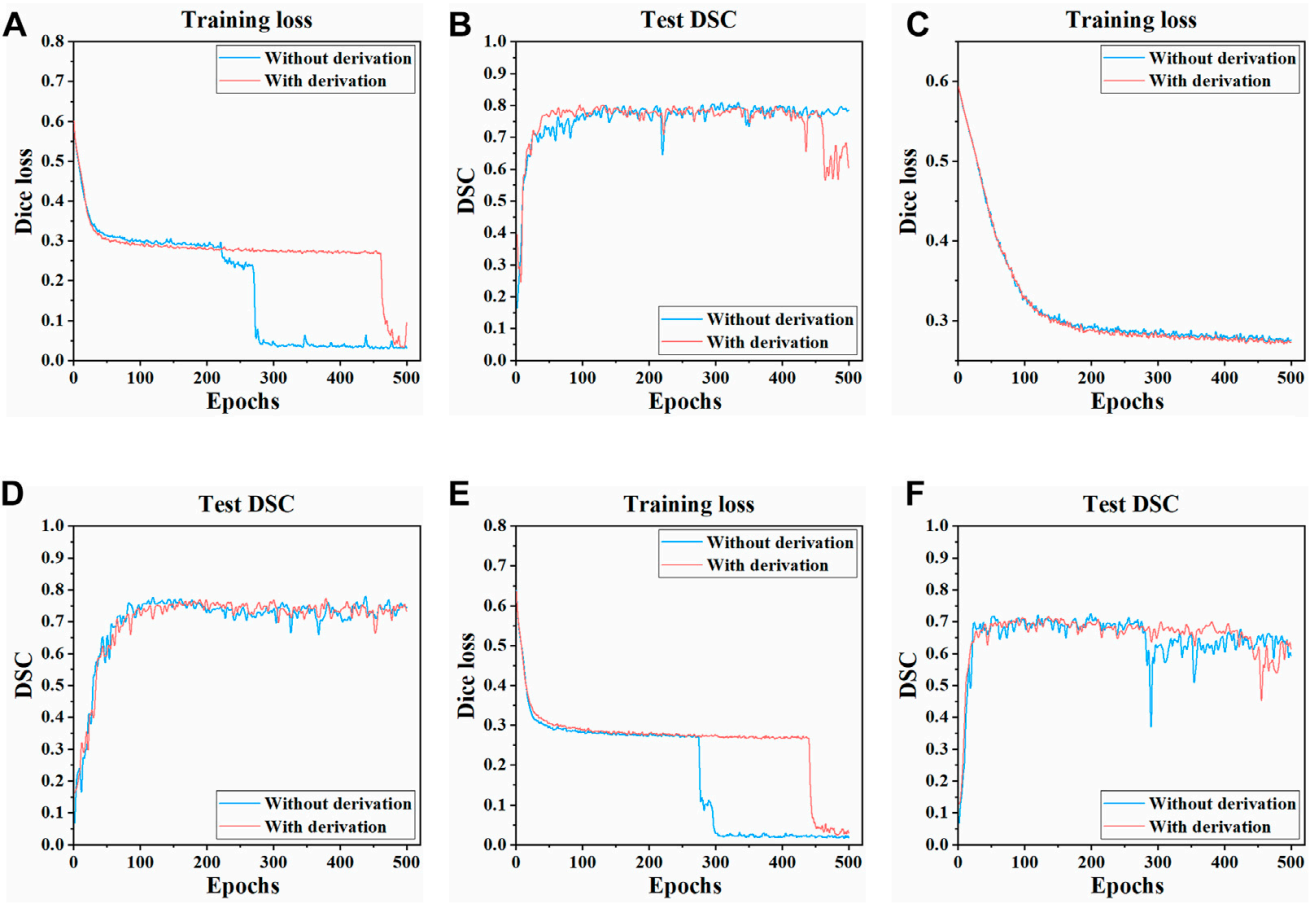
Changes of dice loss value and DSC coefficient in the training process of three models. The changes of the Dice loss on the training set of **(A)** 3D UNet **(C)** VNet, and **(E)** 3D Res-UNet. The changes of the DSC on the test set of **(B)** 3D UNet **(D)** VNet, and **(F)** 3D Res-UNet. The red and blue curves in the figure represent the training process of images with derivation and without derivation, respectively.

The training process and results of three models in two pre-processing methods (with derivation and without derivation) were compared. The Dice loss value change on the training set and the DSC change on the test set during the training process of three models are shown in Figure 4. Compared with the pre-derivation of images, 3D UNet and 3D Res-UNet models can fit and converge faster without derivation while achieving a higher DSC value at the end of training. Besides, the Dice loss value was lower and the DSC value changed more smoothly after convergence. In addition, VNet was not sensitive to the pre-derivation of images, and the Dice loss value and DSC value were similar between derivation and non-derivation during training.

In general, the overall training effect of the three models on the dataset without derivation was better. Therefore, the segmentation effects of the three models on the dataset without derivation were compared, and the subsequent studies in this paper were carried out on the data set without derivation.

#### 3.1.2 Impact of patch size

To explore the influence of different patch sizes on the automatic segmentation results of the 3D UNet model, this study set three different patch sizes (32 × 32 × 32, 48 × 48 × 48, 64 × 64 × 64 voxels). We input samples with different patch sizes into 3D UNet for training and compared the automatic segmentation results of the test set samples, the specific data are shown in Table 1.

**TABLE 1:** Comparison of automatic segmentation results of different patch sizes on 3D UNet.

Patch size	V-Recall	DSC	HD/voxels	AST/s
32 × 32 × 32	0.788 ± 0.153	0.777 ± 0.150	3.934 ± 2.986	0.050
48 × 48 × 48	0.797 ± 0.140	0.818 ± 0.100	3.323 ± 3.212	0.058
64 × 64 × 64	0.809 ± 0.106	0.816 ± 0067	4.621 ± 7.033	0.054

As can be seen from Table 1, the patch size of the sample would affect the segmentation performance of models. When the patch size was 48 × 48 × 48 voxels, the average DSC value on the test set sample was the highest, which was 0.818 ± 0.100, 5.3%, and 0.3% higher than that of 32 × 32 × 32 voxels and 64 × 64 × 64 voxels, respectively. Besides, the HD was also the lowest (3.323 ± 3.212 voxels) when the patch size was 48 × 48 × 48 voxels, which was 15.5% and 28.1% lower than those of 32 × 32 × 32 voxels and 64 × 64 × 64 voxels, respectively. Therefore, when the patch size was 48 × 48 × 48 voxels, 3D UNet had better segmentation performance for IAs.

### 3.2 Performance

#### 3.2.1 Training performance

**FIGURE 5:**
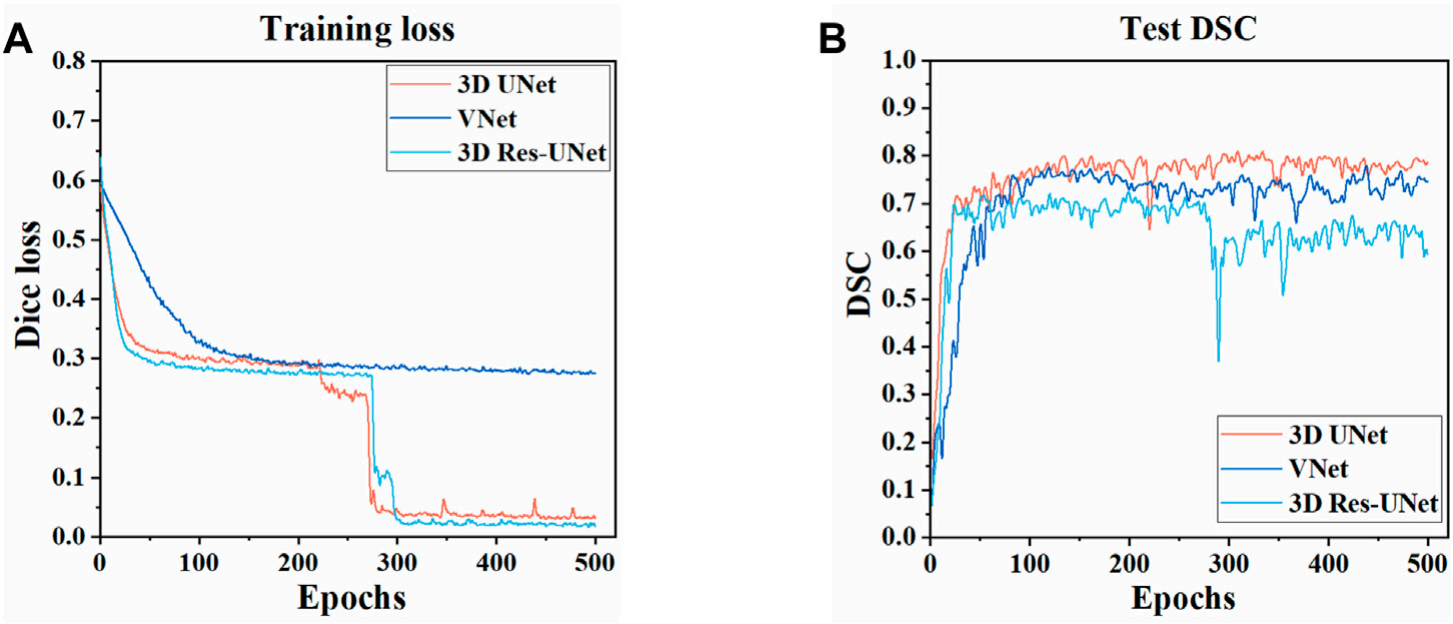
Changes of dice loss value and dice coefficient in the training of three models **(A)** Changes of dice loss on the training set **(B)** Changes of DSC on the test set.

**FIGURE 6:**
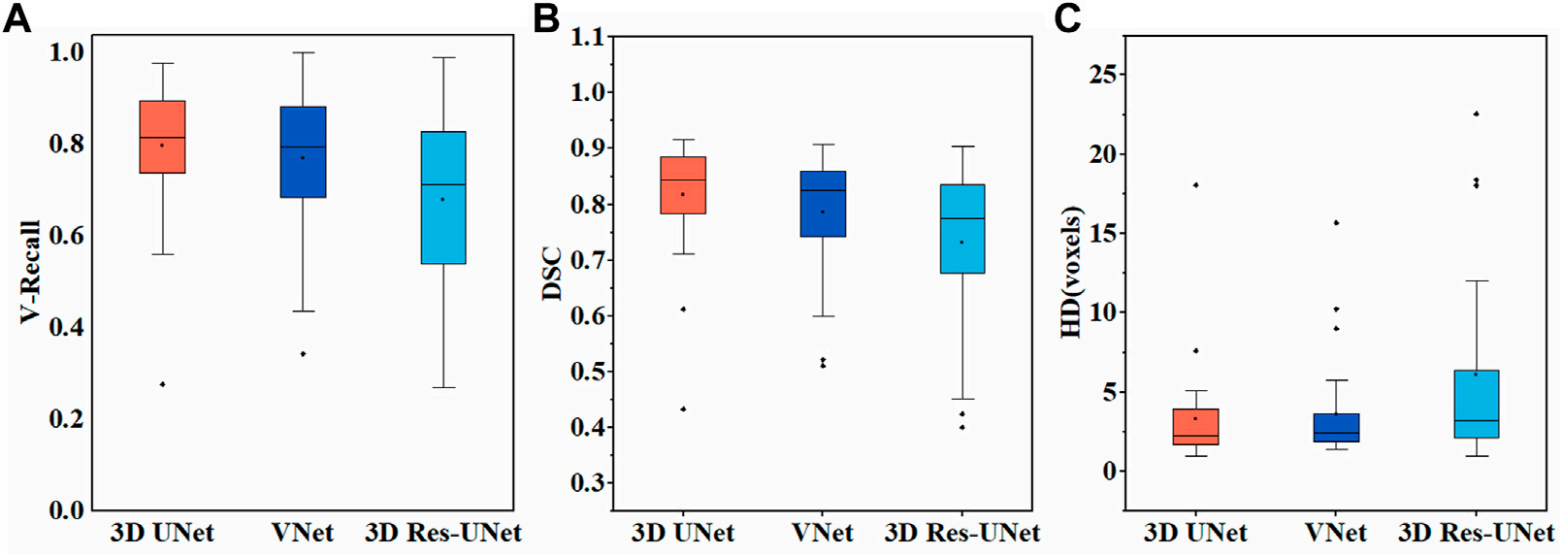
Segmentation results of three models on the test set **(A)** V-Recall **(B)** DSC **(C)** HD.

The three CNNs were trained under the same hyperparameters. Changes in the Dice loss value on the training set and the DSC on the test set during the training of three models are shown in Figures 5, 6. Compared with VNet and 3D Res-UNet, 3D UNet has the fastest convergence rate and the highest DSC value on the test set.

#### 3.2.2 Overall segmentation performance

Figure 6A illustrates the boxplot of the V-Recall of segmentation results on the test set. All the CNNs showed a non-zero voxel-wise recall at the case level, which indicates that all the IAs in each case were identified successfully. Moreover, 3D UNet achieved the highest average V-Recall (79.7%), as well as the highest median V-Recall (81.5%). The average V-Recall of 3D UNet on the test set was 3.5% and 17.3% higher than that of VNet and 3D Res-UNet, respectively. The specific data were listed in Table 2.

**TABLE 2:** Comparison of evaluation performance of three models on test set samples.

Model	V-Recall			DSC			HD/voxels			AST/s
	Average	Median	Range	Average	Median	Range	Average	Median	Range	
3D UNet	0.797 ± 0.140	0.815	0.277–0.976	0.818 ± 0.100	0.844	0.433–0.917	3.323 ± 3.212	2.236	1.000–18.055	0.053
VNet	0.771 ± 0.160	0.795	0.343–1.000	0.786 ± 0.108	0.826	0.511–0.908	3.626 ± 3.167	2.449	1.414–15.684	0.058
3D Res-UNet	0.680 ± 0.205	0.713	0.269–0.989	0.732 ± 0.139	0.776	0.401–0.776	6.080 ± 6.065	3.239	1.000–22.561	0.053

**FIGURE 7:**
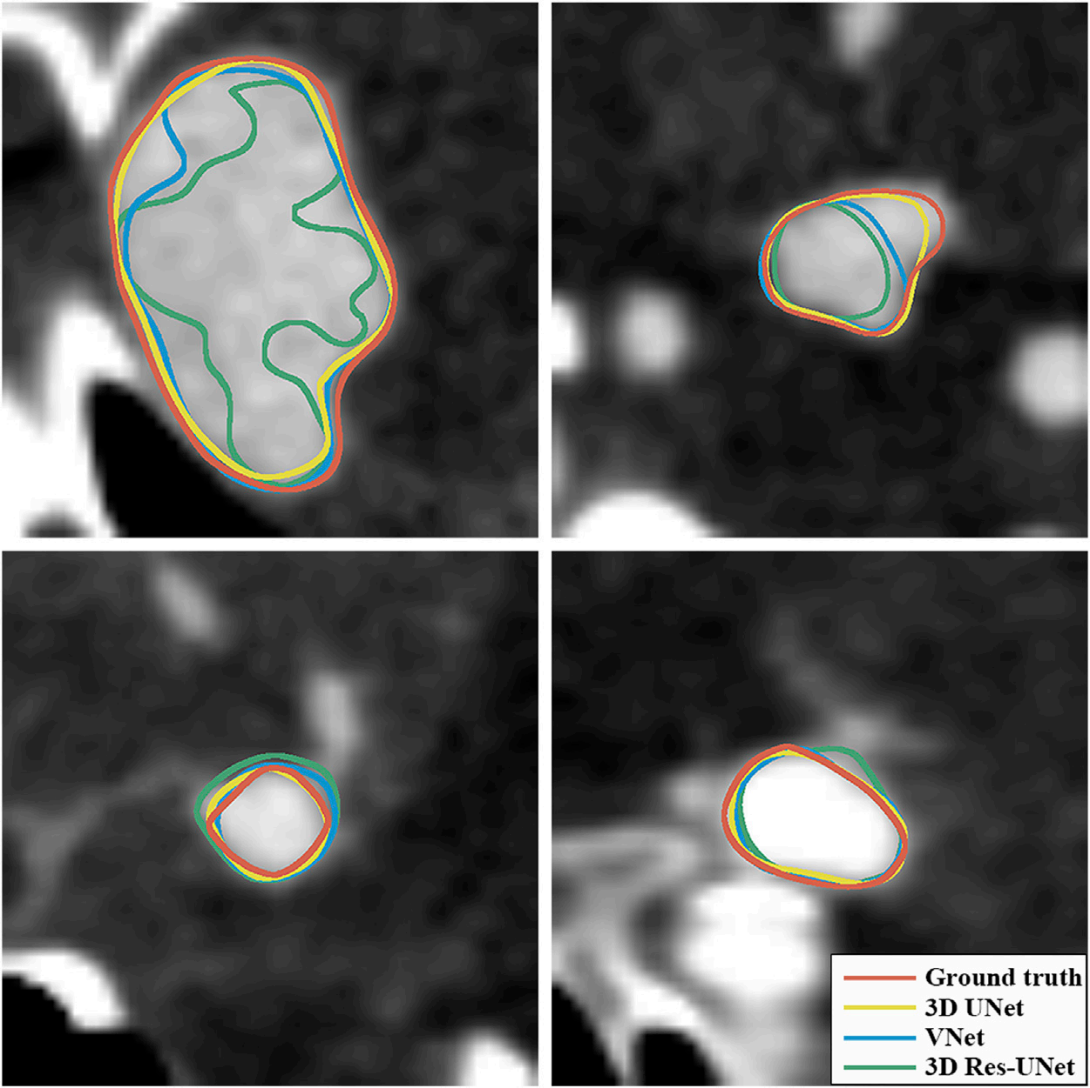
Visualization of segmentation results of three models. The ground-truth annotations are shown in red, and the automatic segmentations of 3D UNet, VNet, and 3D Res-UNet are shown in yellow, blue, and green, respectively.

Figures 6B, C illustrate the boxplots of the DSC and HD of three CNNs’ segmentation results on the test set. The average DSC and HD values of 3D UNet, VNet, and 3D Res-UNet were 0.818 ± 0.100 and 3.323 ± 3.212 voxels, 0.786 ± 0.108 and 3.626 ± 3.167 voxels, 0.732 ± 0.139 and 6.080 ± 6.065 voxels, respectively (Table 2). The average DSC of 3D UNet was 4.1% and 11.7% higher than VNet and 3D Res-UNet, and the average HD was 8.3% and 17.3% lower than that of VNet and 3D Res-UNet, respectively. The segmentation results of three CNNs are illustrated in Figure 7. Among all the compared models, the 3D UNet provides segmentation results most similar to the ground truth.

The AST of 3D UNet and 3D Res-UNet was 0.053 s, which was 8.62% faster than VNet (0.058 s). The specific data of the evaluation performance of the three models on the test set are shown in Table 2.

**FIGURE 8:**
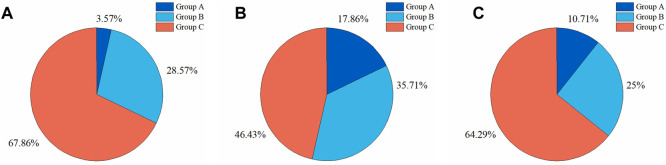
Statistical results of DSC values of segmentation results on the test set. The proportion of segmentation results within different DSC values based on **(A)** 3D UNet **(B)** VNet, and **(C)** 3D Res-UNet. Group A, Group B, and Group C represented samples of DSC between 0.4 and 0.6, 0.6–0.8, and 0.8–1.0, respectively.

To further investigate the DSC distributions of segmentation results, the DSC values of the segmentations in the test set were statistically analyzed. We divided the IA segmentation results into three categories according to the DSC. Group A, Group B, and Group C represented sample groupings of DSC between 0.4 and 0.6, 0.6–0.8, and 0.8–1.0, respectively. For the automatic segmentation based on 3D UNet, 67.86% had a DSC greater than 0.8 (Figure 8A), which was 5.5% and 45.5% higher than that of VNet (Figure 8B) and 3D Res-UNet (Figure 8C), and only 3.57% (1 case) had a DSC between 0.4 and 0.6 (Figure 8A). In the subsequent VNet and 3D Res-UNet, Group C decreased in proportion, while Group A increased in proportion (Figures 8B,C).

#### 3.2.3 Impact of IA size

Sizes and location of IAs are important factors that may affect IA rupture. However, the sensitivity of different models to these factors remains to be studied. Therefore, we compared the segmentation performances of these three models on IAs at different sizes to study the impact of size on model performance.

We divided the IAs of the test set into Group 1, Group 2, and Group 3 according to maximum diameter (D_max_), which were distributed in D_max_ < 5 mm, 5 mm < D_max_ ≤ 10 mm, and D_max_ > 10 mm, respectively. Group 1, Group 2, and Group 3 contained 9, 14, and 4 cases, with an average D_max_ of 3.498, 7.143, and 13.281 mm, respectively.

**FIGURE 9:**
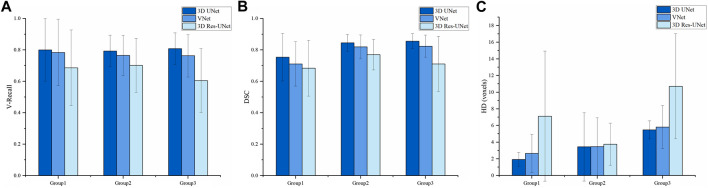
Comparison of IA segmentation results under different sizes **(A)** V-Recall **(B)** DSC **(C)** HD.

Figure 9 illustrates the statistical results of the three models’ mean and standard deviation of the V-Recall, DSC, and HD values. In the groups of IAs of different sizes, 3D UNet achieved the highest average V-Recall (Figure 9A) and DSC (Figure 9B) as well as the lowest average HD (Figure 9C), followed by VNet and 3D Res-UNet. When the maximum diameter (D_max_) of the IAs increased, the V-Recall and DSC values of the segmentation results of the three models increased gradually, and the HD values decreased gradually. It could be seen that the three CNN models had good recognition and segmentation ability for larger IAs. As shown in Table 3, the DSC value of 3D UNet, VNet, and 3D Res-UNet for large aneurysms (D_max_ > 10 mm) in Group 3 was 0.856 ± 0.043, 0.824 ± 0.063, 0.711 ± 0.156, which was 13.5%, 15.9%, 3.9% higher than that of small aneurysms (D_max_ < 5 mm) in Group 1. While in Group 1, 3D UNet still had a DSC of 0.754 ± 0.142, which was 6.0% and 10.2% higher than that of VNet and 3D Res-UNet. V-Recall and HD showed similar dynamics to DSC. Therefore, the segmentation performance of 3D UNet on small aneurysms, general aneurysms, and large aneurysms was relatively balanced, which was better than that of VNet and 3D Res-UNet. VNet performed second. While 3D Res-UNet segmentation results were greatly affected by aneurysm size changes. The specific data were listed in Table 3.

**TABLE 3:** Comparison of segmentation results under IA groups of different sizes.

Group	3D UNet			VNet			3D Res-UNet		
	V-Recall	DSC	HD/voxels	V-Recall	DSC	HD/voxels	V-Recall	DSC	HD/voxels
Group 1	0.800 ± 0.199	0.754 ± 0.142	1.934 ± 0.840	0.784 ± 0.211	0.711 ± 0.133	2.649 ± 2.306	0.686 ± 0.241	0.684 ± 0.168	7.124 ± 7.812
Group 2	0.793 ± 0.100	0.845 ± 0.052	3.445 ± 4.104	0.765 ± 0.127	0.820 ± 0.073	3.472 ± 3.456	0.702 ± 0.171	0.770 ± 0.094	3.754 ± 2.532
Group 3	0.805 ± 0.101	0.856 ± 0.043	5.479 ± 1.085	0.763 ± 0.135	0.824 ± 0.063	5.814 ± 2.568	0.605 ± 0.204	0.711 ± 0.156	10.713 ± 6.295

### 3.3 3D deviation analysis

The 3D deviation analysis results (Table 4) showed that 3D IA reconstruction models based on 3D UNet have the smallest deviation under the above four metrics, while that of 3D Res-UNet has the highest deviation. The max distance of 3D UNet was +1.4760/-2.3854 mm, which was 10.3%/20.1% and 42.2%/48.6% lower than the absolute value of VNet and 3D Res-UNet. The average distance, STD, and RMS of 3D UNet were 0.3480 mm, 0.5978 mm, and 0.7269 mm, which was 28.2% and 56.1%, 22.0% and 22.5%, 24.0% and 35.3% lower than that of VNet and 3D Res-UNet, respectively.

**TABLE 4:** 3D deviation between 3D reconstruction models from CNN-based segmentation and ground truth.

Model	Max distance/mm	Average distance/mm	STD/mm	RMS/mm
3D UNet	+1.4760/−2.3854	0.3480	0.5978	0.7269
VNet	+1.6455/−2.9844	0.4850	0.7666	0.9563
3D Res-UNet	+2.5522/−4.6451	0.7147	0.7711	1.1239

**FIGURE 10:**
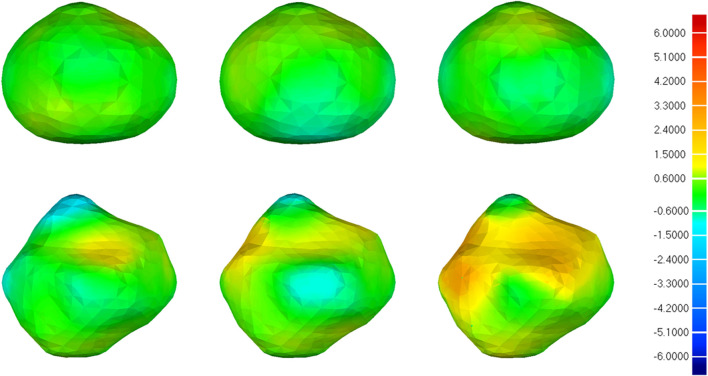
3D deviation analysis results of two IA reconstructed models based on CNN segmentation. The deviation is represented in the reference geometry. The first, second, and third columns represent 3D deviation results for 3D UNet, VNet, and 3D Res-UNet on two IA reconstructed models (upper and lower rows), respectively. The red areas show an overestimation of the reference model and the blue areas indicate an underestimation. Red: +6.000 mm deviation. Green: 0.000 mm deviation. Blue: −6.000 mm deviation.

Figure 10 illustrates the visual distribution of the 3D deviation using the color-coded map to show the differences between CNN-based segmentations compared to the reference. For the IA represented in the first row, all 3D reconstruction models based on three CNN models have minor deviations compared to the reference, and the deviation distribution of the three models is consistent. For the IA in the second row, more yellow areas appear in all 3D reconstruction models, indicating higher deviations. Specifically, 3D IA reconstruction models based on 3D UNet have the smallest deviation.

## 4 Discussion

In this study, three CNN models (3D UNet, VNet, 3D Res-UNet) were constructed and applied to the automatic segmentation of IAs. We compared the automatic segmentation performances of the three models under a small sample size and found that 3D UNet outperformed VNet and 3D Res-UNet. The automatic segmentation results of IAs suggest that the 3D UNet can achieve excellent segmentation quality, which can meet the requirement of the subsequent 3D reconstruction as well as assist clinical diagnosis and treatment. In addition, 3D UNet is also highly time-efficient for a single prediction of less than a second.

### 4.1 Impact of patch size

The patch size plays an important role in getting a precise segmentation of IA lesions, which would affect the negative and positive proportion of the input as well as the consumption of computing resources. Therefore, it is necessary to select the most appropriate patch size according to the specific task for subsequent research. This study compared the impact of three patch sizes (32 × 32 × 32, 48 × 48 × 48, 64 × 64 × 64 voxels) on the IA segmentation using 3D UNet, and found that 3D UNet has better segmentation performance for IAs when the patch size is 48 × 48 × 48 voxels (Table 1). Some previous studies have also shown that a larger patch size doesn’t necessarily mean better (Xu et al., 2019; Essa et al., 2020; Tang et al., 2021).

### 4.2 Performance

#### 4.2.1 Training performance

The convergence and efficiency of CNN training receive much concern in the evaluation of the model since the training is computationally intensive and might affect the segmentation performance (Rehman et al., 2018). In our study, there is no obvious over-fitting phenomenon in the three models. Among them, 3D UNet has the fastest convergence and the highest DSC value on the test set. It might benefit from the use of batch normalization in 3D UNet, which helps the network train faster and achieve higher DSC by reducing internal covariate shifts (Ioffe and Szegedy, 2015; Cicek et al., 2016). Besides, the simple structure of 3D UNet also makes it relatively light and shallow to be available to process the data efficiently under the same hardware conditions (Zou et al., 2018; Lyu et al., 2021).

#### 4.2.2 Segmentation performance

##### 4.2.2.1 Overall segmentation performance

The automatic segmentation and reconstruction quality of IAs have a primary influence on the subsequent hemodynamic analysis. Inaccurate segmentation (especially for important anatomical features, such as aneurysm necks) may result in unrealistic flow patterns and diverging flow parameter values and therefore may even lead to erroneous conclusions (Berg et al., 2018, 2019; Valen-Sendstad et al., 2018).

In this paper, 3D UNet showed excellent IA segmentation performance under a small sample size, which was better than VNet and 3D Res-UNet. In terms of lesion recognition, all the CNNs showed a non-zero V-Recall at the case level, indicating that all the IAs were identified successfully. Among them, 3D UNet achieved the highest average V-Recall of 0.797 ± 0.140, which was 3.5% and 17.3% higher than that of VNet and 3D Res-UNet, respectively (Table 2). This suggested that 3D UNet was more sensitive than the other two models in voxel-wise lesion identification. In terms of lesion segmentation, the automatic segmentation based on 3D UNet reached an average DSC of 0.818 ± 0.100, 4.1%, and 11.7% higher than that of VNet and 3D Res-UNet, as well as an average HD of 3.323 ± 3.212 voxels, 8.3%, and 17.3% lower than that of VNet and 3D Res-UNet (Table 2). These results are comparable to previous studies which used similar image modalities, sample size, and model architecture (Shahzad et al., 2020; Ma and Nie, 2021). Table 5 lists the average DSC achieved in previous studies ranging from 0.53 to 0.8632 (Sichtermann et al., 2019; Shi et al., 2020b; Shahzad et al., 2020; Bo et al., 2021; Ma and Nie, 2021).

**TABLE 5:** Comparison of the segmentation performance of this study with the previous studies.

Study	Networks	Input data format	Number of cases	DSC	HD/voxels	AST/s
This Study	3D UNet	CTA	101	0.818 ± 0.100	3.323 ± 3.212	0.053
Sichtermann et al. (2019)	DeepMedic	3D TOF-MRA	85	0.53 ± 0.30	65.40 ± 18.89	50
Shahzad et al. (2020)	DeepMedic	CTA	253	0.75	—	—
Ma and Nie, (2021)	3D nnUNet	3D CT	132	0.8632	4.97	—
Bo et al. (2021)	GLIA-Net	CTA	1,476	0.579	9.07	25.8
Shi et al. (2020b)	DAResUNet	DSA	1,177	0.75	—	17.6

There could be multiple possible reasons for the better segmentation performance of 3D UNet, for there are differences among the three network architectures. VNet and 3D Res-UNet adopt residual mechanisms based on 3D UNet and use convolution layers instead of pooling layers to perform downsampling. The residual mechanism is mainly proposed to improve the gradient disappearance and gradient explosion in the deep network training through skip connection to realize feature fusion between different layers (He et al., 2016). However, Wang et al. have found that skip connection is not always beneficial, and some inappropriate feature fusion would negatively influence the segmentation performance (Wang et al., 2022). The optimal combination of skip connections should be determined according to the scales and appearance of the target lesions (Wang et al., 2022). Some studies also showed that the effect of the residual mechanism is related to the implementation of specific residual blocks and the input data and pre-processing methods (Naranjo-Alcazar et al., 2019). Our study also demonstrates that adding residual blocks may affect the effectiveness of feature fusion and weaken the IA segmentation performance.

Besides, the performance of CNNs is somewhat different on different datasets. The study by Turečková et al. showed that VNet slightly outperforms 3D UNet on Medical Decathlon Challenge (MDC) Liver dataset, while the trend is opposed in the MDC Pancreas dataset (Turečková et al., 2020). Wang et al. also showed that 3D UNet outperforms VNet in head and neck CT tumor segmentation (Wang G. et al., 2022). Our dataset has high variability in size and shape, similar to those of pancreas and head and neck tumors, resulting in consistent results with the above studies.

##### 4.2.2.2 Impacts of IA size on segmentation performance

Small IAs are a common risk factor for aneurysmal SAH which have a high risk of being missed in clinical screening (Kassell and Torner, 1983; Adams et al., 2000; Weir et al., 2002; Pradilla et al., 2013; Dolati et al., 2015). Whereas, the accurate automatic segmentation of small IAs is still a problem. To verify the impacts of IA size on segmentation performance, we analyzed and found that the performances of the models on small IAs were worse than those on large IAs (similar to other studies (Sichtermann et al., 2019; Shi et al., 2020b; Bo et al., 2021)), and the performance of 3D UNet on small IAs was better than other models. The DSC of 3D UNet, VNet, and 3D Res-UNet for large IAs in Group 3 was 13.5%, 15.9%, and 3.9% higher than that of small aneurysms in Group 1. While in Group 1, the DSC of 3D UNet was 6.0% and 10.2% higher than that of VNet and 3D Res-UNet (Table 3).

Compared with VNet, 3D UNet uses a smaller convolution kernel size which may be more attentive to local IA features and not be overly distracted by the neighborhood when an IA lesion is relatively small and surrounded by many tissues, as Cao et al. demonstrated in their study (Cao et al., 2021).

#### 4.2.3 3D deviation analysis

To evaluate the availability and reliability of the 3D IA models in subsequent hemodynamics, a specific geometric deviation analysis is necessary. Nevertheless, the above metrics (DSC, HD, *etc.*) cannot reflect the accuracy of geometric details, which is important in subsequent mechanical analysis. Thus, we performed the 3D deviation analysis to intuitively evaluate the detailed bias between the CNN-based segmentation and the ground truth. Table 4 shows that 3D UNet had the smallest deviation with a max distance of +1.4760/-2.3854 mm and an average distance of 0.3480 mm. While 3D Res-UNet had the highest deviation, consistent with the trend demonstrated by the above metrics.

What’s more, the visual deviation analysis suggested that high deviations usually occur at the concave and convex areas of irregular IAs (Figure 10). For an IA with regular morphology, the 3D reconstruction models based on three CNN models are more likely to have a small deviation compared with the reference, as shown in the first row of Figure 10. On the contrary, for an IA with irregular morphology, the original concave and convex areas are more likely to be overestimated and underestimated in the 3D reconstruction models, respectively, as shown in the second row of Figure 10. That is because these areas are hard to be segmented accurately by CNN models. Under this premise, we still found that 3D IA reconstruction models based on 3D UNet are more likely to have smaller deviations compared with VNet and 3D Res-UNet.

#### 4.2.4 Segmentation efficiency

Improving the segmentation efficiency is of great significance for meeting clinical needs timely and accelerating the morphological and hemodynamic analysis based on individualized 3days models. As far as we know, automatic segmentation time varies according to different tasks (Patel et al., 2020; Claux et al., 2022). According to some studies, automatic segmentation of IA takes seconds to minutes per case (Sichtermann et al., 2019; Shi et al., 2020b; Bo et al., 2021).

In our study, three CNN models are all highly time-efficient with an AST of less than a second, which is promising for practical use. As the test set uses a smaller patch size (48 × 48 × 48 voxels) than other studies (Sichtermann et al., 2019; Shi et al., 2020b; Bo et al., 2021), the AST of 3D UNet and 3D Res-UNet of an IA is only 0.053s in this study, which is 9.4% less than that of VNet and comparable with the study of Jin et al. (Jin et al., 2019). The longer AST of VNet can be attributed to the use of a larger convolution kernel size, resulting in disproportionally increased expensive computation (Szegedy et al., 2016).

### 4.3 Limitation

The main limitation of this study is related to the small sample size of IAs from a single center, which may result in insufficient diversity of samples. Multicenter study validation should be performed to improve the robustness of results to data from different centers. Besides, all cases in this study were labeled by only one annotator. In the future, cross-validation between different annotators is needed to reduce the impact of individual differences among annotators. Finally, this study only compared three CNN models, and more updated network models can be included for comparison in the future following the same methodology proposed in this study.

## 5 Conclusion

In conclusion, we deployed three CNN models (3D UNet, VNet, 3D Res-UNet) and applied them to the automatic segmentation of IAs. After comparing the automatic segmentation effects of the three models under a small sample size, we found that 3D UNet outperformed VNet and 3D Res-UNet in terms of V-Recall, DSC, and HD. Besides, the 3D Deviation analysis of 3D reconstruction models from CNN-based segmentation also suggested that the segmentation of 3D UNet had the smallest deviation under the above four metrics while that of 3D Res-UNet had the highest deviation, consistent with the above metrics, further demonstrating that 3D UNet is more suitable for IA segmentation than the other two. In terms of segmentation efficiency, three models are all highly time-efficient for a single prediction of less than a second in this study, which is promising for practical use in the real-time diagnosis of cerebral hemorrhage and treatment of IAs. This can greatly facilitate current large-scale CTA-based precise patient-specific modeling and analysis studies in healthcare.

The study of IA is vital for the health of the public. In the future, beyond the automatic detection and segmentation, predicting the rupture of IAs according to the morphological and hemodynamic analysis based on individualized 3D models will be worth exploring. Therefore, 3D UNet can not only assist clinicians in the diagnosis of IAs but can also encourage more implementations of artificial intelligence in healthcare.

## Data Availability

The original contributions presented in the study are included in the article/supplementary material, further inquiries can be directed to the corresponding authors.
